# Lorcaserin *vs.* Phentermine among non-surgical and surgical obese patients: Anthropometric, glycemic, lipid, safety and cost outcomes

**DOI:** 10.1016/j.amsu.2019.07.024

**Published:** 2019-07-13

**Authors:** Wahiba Elhag, Walid El Ansari, Sama Abdulrazzaq, Mohamed Elsherif, Isra Mustafa

**Affiliations:** a Department of Bariatric Surgery/Bariatric Medicine, Hamad General Hospital, Hamad Medical Corporation, Doha 3050, Qatar; b Department of Surgery, Hamad General Hospital, Hamad Medical Corporation, Doha 3050, Qatar; c College of Medicine, Qatar University, Doha, Qatar; d School of Health and Education, University of Skövde, Skövde, Sweden

**Keywords:** Obesity, Lorcaserin, Phentermine, Weight regain, Bariatric surgery, Lipid profile, Glycemic parameters

## Abstract

**Background:**

To evaluate effectiveness, safety, and costs of Lorcaserin *vs.* phentermine among obese non–surgical and surgical patients (post bariatric surgery).

**Methods:**

This retrospective study retrieved charts of all patients (January 2013–June 2016) who received Lorcaserin or phentermine for 3 months. The study assessed anthropometric, glycemic, and lipid changes, as well as side effects and cost of medications among overweight and obese non-surgical (n = 83) and surgical patients (n = 46). These two patient groups were compared using Chi-square (χ2) and unpaired‘t’ test for qualitative and quantitative variables respectively.

**Results:**

At 3 months, among the non-surgical group, Phentermine patients had greater percentage of total weight loss (TWL%) (7.65 ± 8.26 *vs.* 2.99 ± 3.72%, P = 0.003), and greater BMI reduction (−3.16 ± 3.63 *vs.* −1.15 ± 1.53 kg/m^2^, P = 0.003) than Lorcaserin. Within the surgical group, Lorcaserin patients had significantly smaller TWL% (1.86 ± 5.06 *vs.* 7.62 ± 9.80%, P = 0.012), and smaller BMI reduction (−0.74 ± 1.80 *vs.* −3.06 ± 4.08 kg/m^2^, P = 0.012) than Phentermine. Lorcaserin exhibited significant total cholesterol and LDL improvements only among surgical patients with significant weight reduction (≥5% TW). Both medications were not associated with glycemic improvements among non-surgical and surgical groups. Phentermine had slightly more side effects but was less expensive.

**Conclusions:**

Among both patient groups, phentermine was more effective in achieving weight loss. Lorcaserin showed dyslipidemia improvements only among surgical patients who achieved significant weight reduction. Anti-obesity medications as part of weight management programs can result in weight loss among non-surgical and surgical patients, or halt weight regain among surgical patients. This is the first study to evaluate the effectiveness and safety of two anti-obesity medications (lorcaserin vs. phentermine) among two distinct obese patient groups, non-surgical and surgical patients.

## Highlights

• First study to evaluate effectiveness and safety of lorcaserin vs. phentermine in obese non-surgical and surgical patients.
• Among both patient groups, phentermine was more effective in achieving weight loss than lorcaserin.
• Dyslipidemia improvements was only achieved in surgical patients who achieved significant weight reduction with lorcaserin.
• Both medications were not associated with glycemic improvements among non-surgical and surgical groups.
• Phentermine had slightly more side effects but was less expensive.

## 1 Background

Obesity is a pathophysiological driver of cardiovascular morbidity and mortality. It is also linked to type 2 diabetes, hypertension and hyperlipidemia [1,2]. Modest weight loss [≥ 5% Total Weight (TWL%)] achieved by lifestyle modifications and anti-obesity medications may improve such obesity-related comorbidities [3,4]. Anti-obesity medications are indicated for those with BMI ≥27 and ≥ 1 comorbidity, or those with BMI ≥30 who are not candidates for bariatric surgery [5]. They may also be a safer alternative to complicated revisional surgeries for bariatric surgery patients who do not achieve adequate weight loss (5–15% of patients) or regain weight after surgery (5.7–75% of patients) [[6], [7], [8], [9]]. Lorcaserin and Phentermine are commonly used anti-obesity medications: the former is a selective serotonin 2C receptor agonist that increases satiety through the proopiomelanocortin system [10]; the latter is an appetite suppressant that inhibits the reuptake of nor-epinephrine in the hypothalamus [10].

It is important to assess the efficacy of multiple anti-obesity medications on different patient populations [11,12]. Previous studies assessed only one medication [[13], [14], [15]] or one patient population i.e. non-surgical (no previous bariatric surgery) or surgical patients (with previous bariatric surgery and subsequent weight regain/inadequate weight loss) [8,16,17]. The one study that compared Lorcaserin *vs.* Phentermine among non-surgical and surgical patients reported only anthropometric outcomes, did not assess cardiovascular outcomes but side effects or costs [11]. To the best of our knowledge, no study compared Lorcaserin *vs.* Phentermine among non-surgical and surgical patients across a range of cardiovascular and anthropometric outcomes, side effects, and costs.

Therefore, this retrospective study compared the effectiveness and safety of Lorcaserin *vs.* Phentermine at 3 months, among two distinct groups: non-surgical and surgical patients. The effectiveness measures included changes in three anthropometric [weight, BMI, total weight loss% (TWL%)], two glycemic [hemoglobin A1c, fasting blood glucose (HBA1c, FBS)] and four lipid [(total cholesterol, low density Lipoprotein, high density lipoprotein, triglycerides (TC, LDL, HDL, TG)] parameters. The study also evaluated the side effects and costs of both medications.

## 2 Material and methods

### 2.1 Ethics and sittings

The Medical Research Centre at Hamad Medical Corporation (HMC) approved this retrospective study (IRB, Protocol #17193/17) which was conducted at the Bariatric Clinic, Hamad General Hospital, Doha, Qatar.

### 2.2 Registration

This study is registered with the Research Registry [18] (Research Registry UIN: research registry 4801.

### 2.3 Study design

The study is a retrospective record review.

### 2.4 Procedures and data collection

Using medical charts and electronic records, Lorcaserin or Phentermine prescription data was retrieved for both patient groups (non-surgical and surgical). Baseline and 3 months post-treatment values were retrieved, and change was calculated (value at 3 months minus baseline value) for the following: Anthropometric parameters (weight, BMI, and TWL%), TWL%, calculated as: [baseline weight (before treatment) –follow-up weight (at 3 months)]/baseline weight × 100; lipid profile parameters (TC, LDL, HDL, TG); and, Glycemic parameters (FBS, HbA1c). Standard referential values were used for the assays of these parameters in the blood. Data on reported side effects included potential gastrointestinal, cardiovascular, and neurological side effects.

### 2.5 Inclusion and exclusion criteria

All patients who received Lorcaserin or phentermine for 3 months during the study period (January 2013–June 2016, N = 198) were eligible to be included. The inclusion criteria included age (≥18 years old) and BMI (≥30 or ≥ 27 with comorbidities). Patients for whom 3 months follow-up data was unavailable (n = 13), on combination medications (n = 54), or had endoscopic procedures (intra-gastric balloon/intra-gastric Botox injection, n = 2) were excluded. The remaining 129 patients included in the final analysis comprised 83 non-surgical patients; and 46 surgical patients who were receiving Lorcaserin or Phentermine due to weight regain (n = 34) or inadequate weight loss (n = 12). Weight regain is defined as an increase of >10 kg from nadir [19]. Inadequate weight loss is defined as < 50% excess weight loss (EWL% at 18 months after surgery [20].

### 2.6 Standard care at the Bariatric Clinic

All patients had initial screening blood tests and baseline ECG. The choice of anti-obesity medication is individualized based on patient preference, the drug's contraindications and side effects, as well as their potential interactions with the patient's existing medication and co-morbidities. Lorcaserin was given 10 mg twice daily, while Phentermine given 37.5 mg once daily. All patients were followed up by a multidisciplinary team of bariatric physicians, dietitians, and physiotherapists. Surgical patients were additionally followed up by bariatric surgeons at 1, 3, 6, 12 months, and semiannually thereafter. All patients had follow-up blood tests at 3 months. Dieticians and physical therapists individually counseled all patients on routine dietary intake and physical activity. Dietary counseling encouraged patients to follow a low-calorie high protein (1000–1200 calories/day) diet, and protein supplements were provided as meal replacement. Physical activity was based on patient's age, medical condition, physical abilities, and preference. Patients were encouraged to exercise for 150–300 min per week.

### 2.7 Statistical analysis

Statistical analysis was performed using the statistical software SPSS 22.0 (SPSS Inc. Chicago, IL). Two-tailed P values were presented with P values < 0.05 considered statistically significant. Categorical and continuous values were expressed as frequency (percentage) and mean ± SD. Descriptive statistics summarized the demographic, anthropometric, glycemic, lipid, and clinical characteristics. The Kolmogorov-Smirnov (K–S) test assessed the data normality. The primary outcome was to assess and compare the effectiveness (anthropometric, glycemic, lipid, clinical changes) and safety (side effects) of two anti-obesity medications (Lorcaserin vs. Phentermine) at 3 months among two independent weight-management groups: non-surgical patients and surgical patients. Associations between two or more qualitative variables were assessed using chi-square (χ2) test and Fisher Exact test or Yates corrected chi-square, as appropriate. Quantitative data between the two independent groups was analyzed using unpaired ‘t’ test (Mann Whitney *U* test for non-normal data).

### 2.8 Reporting

This study is reported in line with the STROCSS guidelines [21].

## 3 Results

Table 1 depicts the weight history of the 46 surgical patients, noting that 40 had laparoscopic sleeve gastrectomy, 4 had Roux-en Y gastric bypass, and 2 had gastric band (data not presented).

**Table 1:** Weight history of Surgical patients (*N* = 46).

	Mean	SD
Pre-op weight (kg)	94.30	16.45
Pre-op weight BMI (kg/m^2^)	37.16	5.73
Nadir weight (kg)	83.98	14.98
Nadir BMI (kg/m^2^)	32.97	4.93
Surgical weight loss (kg)	34.74	16.27
Total weight loss % (TWL%)	29.58	14.67
Time since surgery (years)	3.5	1.84
Weight nadir to program weight (kg)	10.49	7.81
% weight regain from nadir to program weight	35.08	26.90

Surgical patients included those with inadequate weight loss or weight regain; SD: standard deviation

Table 2 depicts the baseline characteristics of the non–surgical and surgical patients by anti-obesity medication. Significant differences in the effects of Lorcaserin vs. Phentermine were observed only among the non–surgical patients; Lorcaserin patients had lower mean LDL but a higher proportion of dyslipidemia than Phentermine patients. There were no other differences across the other variables.

**Table 2:** Baseline characteristics of the patient groups by anti-obesity medication.

Demographic and Anthropometric	Non-surgical patients*			Surgical patients**		
	Phentermine	Lorcaserin		Phentermine	Lorcaserin	
	(N = 48)	(*N* = 35)		(*N* = 19)	(N = 27)	
	Mean ± SD	Mean ± SD	*P*	Mean ± SD	Mean ± SD	*P*
Age, y	40.91 ± 10.96	42.05 ± 10.71	0.638	45.05 ± 9.44	43.81 ± 10.39	0.682
Gender, *N* (%)			0.179			0.772
Male	11 (22.9)	4 (11.4)		1 (5.3)	2 (7.4)	
Female	37 (77.1)	31 (88.6)		18 (94.7)	25 (92.6)	
Height, m	1.61 ± 0.08	1.59 ± 0.08	0.172	1.59 ± 0.07	1.58 ± 0.07	0.695
Weight, kg	100.20 ± 20.88	94.55 ± 20.83	0.227	94.84 ± 18.18	93.91 ± 15.46	0.852
BMI, kg/m^2^	38.27 ± 7.57	37.17 ± 7.32	0.512	36.98 ± 5.27	37.28 ± 6.13	0.859
EW, kg	34.53 ± 19.74	30.95 ± 18.77	0.409	30.93 ± 14.83	30.68 ± 14.81	0.956
Glycemic						
FBS, mmol/l	6.02 ± 3.89	5.69 ± 1.69	0.640	4.78 ± 0.84	4.70 ± 0.75	0.728
HbA1c, %	6.06 ± 1.50	5.60 ± 0.75	0.110	5.64 ± 0.86	5.50 ± 0.47	0.494
Lipid profile, mmol/l						
TC	4.96 ± 0.74	4.63 ± 0.78	0.060	5.14 ± 0.99	5.11 ± 0.70	0.916
LDL	3.08 ± 0.67	2.73 ± 0.65	0.021	3.08 ± 0.85	3.11 ± 0.60	0.886
HDL	1.27 ± 0.30	1.35 ± 0.34	0.285	1.61 ± 0.42	1.43 ± 0.27	0.098
TG	1.43 ± 1.21	1.25 ± 0.53	0.403	0.97 ± 0.42	1.17 ± 0.50	0.186
Clinical, *n* (%)	*n* (%)	*n* (%)	*P*	*n* (%)	*n* (%)	*P*
Prediabetes			0.061			0.225
Yes	9 (18.8)	13 (37.1)		0 (0)	2 (7.4)	
No	39 (81.3)	22 (62.9)		19 (100)	25 (92.6)	
Diabetes			0.425			0.175
Yes	9 (18.8)	4 (12.1)		6(31.6)	4 (14.8)	
No	39 (81.3)	29 (87.9)		13 (68.4)	24 (85.2)	
Hypertension			0.674			0.928
Yes	9 (18.8)	5 (15.2)		3 (15.8)	4 (14.8)	
No	39 (81.3)	28 (84.8)		16 (84.2)	25 (85.2)	
Dyslipidemia			0.010			0.321
Yes	9 (18.8)	15 (45.5)		4 (21.1)	9 (34.6)	
No	39 (81.3)	18 (54.5)		15 (78.9)	17 (65.4)	

Patient group: on medical weight management, *without previous bariatric procedure or **with previous bariatric procedure, SD standard deviation, BMI Body Mass Index, EW excess weight, FBS fasting blood sugar, HBA1c glycosylated hemoglobin A1c, TC Total Cholesterol, LDL low density Lipoprotein, HDL high density lipoprotein: high density lipoprotein, TG triglycerides.

Table 3 shows the changes at 3 months. Within the non–surgical group, Phentermine patients lost more weight (−8.42 ± 9.69 vs. −2.98 ± 4.15 kg, P = 0.003), had a greater percentage of weight loss (7.65 ± 8.26% vs. 2.99 ± 3.72%, P = 0.003), and had greater BMI change (3.16 ± 3.63 vs. 1.15 ± 1.53 kg/m^2^, P = 0.003) than Lorcaserin patients. On the other hand, Lorcaserin patients had significantly lower mean LDL than Phentermine patients, although such significant differences were already present at baseline. No other significant changes were observed in the non-surgical group. Within the surgical group, Lorcaserin patients had significantly less weight reduction (−1.81 ± 4.54 vs. −7.68 ± 10.32 kg, P = 0.012), smaller percentage of weight change (1.86% vs. 7.62%, P = 0.012), and lower reduction in BMI (−0.74 ± 1.80 vs. −3.06 ± 4.08 kg/m^2^, P = 0.012) than Phentermine patients. There were no other significant changes observed between the two anti-obesity medications in the surgical group.

**Table 3:** Changes at 3 months by patient group.

	Non-surgical patients*			Surgical patients**		
	Phentermine (*N* = 48)	Lorcaserin (*N* = 35)		Phentermine (*N* = 19)	Lorcaserin (*N* = 27)	
Anthropometric	Mean ± SD	Mean ± SD	*P*	Mean ± SD	Mean ± SD	*P*
Weight, kg	91.78 ± 16.90	91.56 ± 19.36	0.958	87.16 ± 18.03	92.06 ± 15.24	0.322
Change from Baseline, kg ^*a*^	8.42 ± 9.69	2.98 ± 4.15	0.003	7.68 ± 10.32	1.81 ± 4.54	0.012
TWL%^*b*^	7.65 ± 8.26	2.99 ± 3.72	0.003	7.62 ± 9.80	1.86 ± 5.06	0.012
BMI, kg/m^2^	35.11 ± 6.39	36.02 ± 6.94	0.537	33.92 ± 4.66	36.54 ± 5.90	0.114
Change from Baseline	3.16 ± 3.63	1.15 ± 1.53	0.003	3.06 ± 4.08	0.74 ± 1.80	0.012
TWL (%)						
< 5%	11(22.9)	24(68.6)	<0.0001	5(26.3)	13 (48.1)	0.135
5–9.9%	10 (20.8)	3 (8.6)	0.129	4 (21.1)	7 (25.9)	0.703
10–19.9%	14 (29.2)	4 (11.4)	0.493	3 (15.8)	1 (3.7)	0.152
≥ 20%	8 (8.3)	0 (0)	0.080	3 (15.8)	0 (0)	0.033
Weight gain	6 (12.5)	4 (11.4)	0.882	4 (21.1)	6 (22.2)	0.925
Glycemic						
FBS, mmol/l	5.87 ± 2.62	5.51 ± 1.20	0.541	4.59 ± 0.62	4.88 ± 0.78	0.247
Change from Baseline	0.29 ± 2.29	0.52 ± 1.50	0.679	−0.03 ± 0.81	−0.28 ± 0.52	0.317
HBA1C %	5.94 ± 1.19	5.61 ± 0.88	0.279	5.59 ± 0.70	5.42 ± 0.60	0.477
Change from Baseline	0.15 ± 0.37	0.10 ± 0.77	0.797	0.15 ± 0.52	0.10 ± 0.23	0.720
Lipid profile, mmol/l						
TC	4.91 ± 0.72	4.66 ± 0.69	0.189	5.03 ± 1.10	4.81 ± 0.77	0.507
Change from Baseline	0.22 ± 0.62	−0.00 ± 0.80	0.231	−0.01 ± 0.53	0.30 ± 0.60	0.123
LDL	3.09 ± 0.81	2.62 ± 0.56	0.018	2.95 ± 0.90	2.85 ± 0.61	0.698
Change from Baseline	0.15 ± 0.79	0.03 ± 0.68	0.567	0.04 ± 0.38	0.19 ± 0.50	0.360
HDL	1.38 ± 0.49	1.36 ± 0.38	0.873	1.61 ± 0.33	1.38 ± 0.29	0.041
Change from Baseline	−0.08 ± 0.46	0.03 ± 0.30	0.246	−0.03 ± 0.22	0.08 ± 0.12	0.084
Triglycerides	1.28 ± 0.78	1.30 ± 0.61	0.924	0.91 ± 0.44	1.29 ± 0.64	0.063
Change from Baseline	0.14 ± 0.65	0.06 ± 0.49	0.590	0.02 ± 0.29	−0.06 ± 0.41	0.474

Patients: on medical weight management *without previous bariatric procedure or **with previous bariatric procedure, ^*a*^mean value at 3 months minus mean baseline value, ^*b*^change from baseline divided by baseline weight x 100, TWL% percentage of total body weight loss, BMI body mass index, FBS fasting blood sugar, HbA1c glycosylated hemoglobin A1C, TC Total Cholesterol, LDL low density Lipoprotein, HDL high density lipoprotein, TG triglycerides.

Table 4 illustrates the analysis of the changes at 3 months for the subgroup of non–surgical and surgical patients who had significant weight loss (≥5% of TW). Only among the surgical group, Lorcaserin patients had reduction in TC (−0.85 ± 0.51 vs. −0.02 ± 0.33 mmol/l, P = 0.003) and LDL (0.57 ± 0.39 vs. 0.02 ± 0.25 mmol/l, P = 0.008) levels when compared with Phentermine patients. There were no other significant changes observed between Phentermine vs. Lorcaserin in the surgical group or non-surgical group in terms of lipid profile or glycemic parameters.

**Table 4:** Changes at 3 months for patients with significant weight loss ≥5% of TW.

	Non-surgical patients*			Surgical patients**		
	Phentermine (*N* = 31)	Lorcaserin *(N* = 7)		Phentermine (*N* = 10)	Lorcaserin (*N* = 8)	
Glycemic Mean	Mean ± SD	Mean ± SD	P	Mean ± SD	Mean ± SD	*P*
FBS, mmol/l	5.88 ± 2.62	5.20 ± 1.21	0.544	4.61 ± 0.62	4.87 ± 0.51	0.402
Change from Baseline	0.58 ± 2.60	0.68 ± 1.13	0.930	0.22 ± 0.38	−0.04 ± 0.53	0.022
HBA1C %	6.02 ± 1.26	5.52 ± 0.60	0.404	5.56 ± 0.50	5.31 ± 0.38	0.309
Change from Baseline	0.08 ± 0.33	0.06 ± 0.59	0.906	0.02 ± 0.33	0.18 ± 0.16	0.311
Lipid profile, mmol/l (mean ± SD)						
TC	4.88 ± 0.77	4.77 ± 0.64	0.756	5.12 ± 1.05	4.50 ± 0.53	0.188
Change from Baseline	0.37 ± 0.69	0.36 ± 0.32	0.966	0.02 ± 0.33	0.85 ± 0.51	0.003
LDL	2.97 ± 0.74	2.60 ± 0.40	0.303	3.07 ± 1.00	2.65 ± 0.57	0.347
Change from Baseline	0.35 ± 0.63	0.40 ± 0.74	0.897	0.02 ± 0.25	0.57 ± 0.39	0.008
HDL	1.28 ± 0.26	1.29 ± 0.25	0.997	1.52 ± 0.23	1.30 ± 0.23	0.104
Change from Baseline	−0.003 ± 0.29	0.01 ± 0.20	0.902	0.06 ± 0.18	0.06 ± 0.09	0.964
Triglycerides	1.40 ± 0.91	1.64 ± 0.98	0.615	0.96 ± 0.50	1.21 ± 0.67	0.430
Change from Baseline	0.16 ± 0.61	−0.03 ± 0.61	0.540	0.08 ± 0.31	0.09 ± 0.21	0.913

Patient group: on medical weight management *without previous bariatric procedure or **with previous bariatric procedure, FBS fasting blood sugar, HbA1c glycosylated hemoglobin A1C, TC Total Cholesterol, LDL low density Lipoprotein, HDL high density lipoprotein, TG triglycerides.

In terms of safety, Table 5 shows that the most common reported side effects were palpitations for Phentermine (6%) and headache for Lorcaserin (5.9%). Table 6 depicts the comparative cost analysis of Phentermine vs. Lorcaserin. For each patient, a 30-day supply of Phentermine costs USD 18.28 and USD 283.82 for Lorcaserin [22]. Based on a 3-months treatment regime, savings of USD 49280.76 would have been achieved had Phentermine been prescribed to all Lorcaserin patients (assuming none of the Lorcaserin patients had any contraindications to Phentermine). Extrapolating further, Table 6 also shows the potential savings that could have resulted when applying the costs of the two anti-obesity medications to the findings of Grabarczyk et al. in a recent larger study of a longer duration (12 months, 298 patients) [12]. If Grabarczyk et al. would have prescribed Phentermine to all their Lorcaserin patients for the same 12-month period [12], their potential savings would have amounted to USD 949571.

**Table 5:** Side effects by anti-obesity medication.

Side effect	Phentermine *N* (%)	Lorcaserin *N* (%)
Headache	0 (0)	3 (5.9)
Blurred Vision	0 (0)	1 (2)
Dizziness	1 (1.5)	1 (2)
Dry Mouth	0 (0)	0 (0)
Constipation	2 (3)	0 (0)
Drowsiness	1 (1.5)	–
Depression	0 (0)	0 (0)
Numbness	0 (0)	–
Restlessness	0 (0)	–
Insomnia	3 (4.5)	–
Nervousness	1 (1.5)	–
Palpitation	4 (6)	–

All cells represent *N* (%), — not applicable.

**Table 6:** Comparative cost analysis by anti-obesity medication.

Variable	Current study* (3 months)			Previous study Grabarczyk** (12 months)		
	Lorcaserin	Phentermine	Saving	Used Lorcaserin	If used Phentermine	Saving
Total number of patients	62	67	–	298	298	–
Duration of treatment	3 m	3 m	–	12 m	12 m	–
Cost per month per patient	283.82	18.28	265.54	283.82	18.28	265.54
Total cost per patient	851.46 for 3 m	54.84 for 3 m	796.62 for 3 m	3405.84 for 12 m	219.36for 12 m	3186.48
Total cost for period (USD) ^*a*^	52790.52	3509.76	49280.76	1.015 M	65369.28	949571.04

*Calculated for current study period (3 months), ** Calculated for their prescription period (1 year), All costs presented in USD, — not applicable, *m* months, M: million, ^*a:*^ cost for 62 patients.

Table 7 summarizes all the study findings among non–surgical and surgical patients.

**Table 7:** Summary of Changes at 3 months by patient group.

	Non-surgical patients*	Surgical patients**
Parameter	Phentermine *vs.*Lorcaserin (48 *vs.*35)	Phentermine *vs.*Lorcaserin (19 *vs.*27)
Anthropometric mean (SD)		
Weight		
Actual weight	No difference	No difference
Weight change^*a*^ and TWL%^*b*^	Phentermine associated with greater weight reduction and more TWL%	Phentermine associated with greater weight reduction and more TWL%
BMI		
BMI level	No difference	No difference
BMI change	Phentermine associated with more BMI reduction	Phentermine associated with more BMI reduction
TWL (%)	No difference at all levels of TWL%, except Lorcaserin associated with higher percentages of patients with lower (<5%) TWL%	No difference at all levels of TWL%, except Phentermine associated with higher percentages of patients with higher (≥20%) TWL%
Weight gain	No difference	No difference
Glycemic (FBS, HBA1C)	No difference in FBS or HBA1C levels	No difference in FBS or HBA1C levels
Lipids (TC, LDL, HDL, TG)		
Actual levels	No difference, except Lorcaserin associated with lower LDL level	No difference except Phentermine associated with higher HDL level
Level Change	No difference for all lipids	No difference for all lipids
Side effects	Phentermine slightly more	Lorcaserin slightly more
Cost	Phentermine less expensive	Phentermine less expensive

*Without previous bariatric procedure, **With previous bariatric procedure, ^*a*^mean value at 3 months minus the mean baseline value, ^*b*^change from baseline divided by the baseline weight x 100, TWL% percentage of total body weight loss, BMI Body Mass Index, FBS fasting blood sugar, HbA1c glycosylated hemoglobin A1C, TC Total Cholesterol, LDL low density Lipoprotein, HDL high density lipoprotein, TG triglycerides.

## 4 Discussion

Studies on the effectiveness and safety of anti-obesity medications for weight loss and cardiovascular risk factors remain limited [15]. The current study compared the effectiveness and safety of Phentermine vs. Lorcaserin among non-surgical and surgical patients. The main findings are that at 3 months, among the non-surgical group, Phentermine patients significantly lost more weight, and had higher TWL% and BMI change when compared to Lorcaserin patients. Further analysis of the patient subgroup that achieved significant weight reduction (≥5% TW) showed that only in the surgical group, Lorcaserin patients had significant changes in TC and LDL when compared to Phentermine. However, the weight reductions observed with both anti-obesity medications were not associated with any significant improvement in glycemic control in both groups. Both medications were well tolerated with few side effects. Phentermine was less expensive than Lorcaserin (savings = $796.62 USD per 3-month regime/patient).

In terms of anthropometric parameters among the non-surgical group, the current study found that at 3 months, Phentermine patients exhibited significantly higher TWL% when compared with Lorcaserin (7.65% vs. 2.99%, P = 0.03). This is contrary to other studies that reported similar TWL% (3.6%) between Lorcaserin and Phentermine [12]. Most Phentermine studies are small and outdated (e.g. [23,24]). One recent study used a diffuse controlled release Phentermine formulation on a small sample (n = 37) and reported that 95.8% of their patients achieved WL ≥ 5%. That was greater than the 58.3% observed by the current study (data not presented), which is likely explained by the diffuse controlled release formulation used [25]. As for Lorcaserin patients, 8.6% lost ≥5% and 11.4% lost ≥10% of TW. These results are lower than other studies (47.3% lost ≥5% and 22.6% lost ≥10% of TW respectively) [26], probably due to their longer study duration (1 year) [25], or generally due to psycho-behavioral and nutritional factors [27]. As for the anthropometric parameters among the surgical group, at 3 months significantly more Phentermine patients (31.6%) achieved ≥10% TWL% compared to Lorcaserin patients (3.7%) patients. These findings support other research suggesting that Phentermine could be useful among surgical patients with weight regain [8,19]. However, we are unable to directly compare the Lorcaserin findings with other studies because other studies did not report weight loss outcomes individually for Lorcaserin [11,28]. In terms of surgical weight history, the average time for weight regain post bariatric surgery was shorter for patients in the current study for weight regain after (3.5 years) compared with other reports (6.1 years) [11] . Despite the shorter duration, the average weight regain in the current study was 35% of the minimum weight loss achieved post-surgery, similar to the 34% reported by others [11]. This is likely attributed to several factors: technical/anatomical, physiological, psychosocial, and lifestyle/behaviour [9].

In terms of lipid profile among the non-surgical group, at 3 months, both anti-obesity medications did not lead to significant improvement. This observation agrees with other studies on Lorcaserin [15], yet it contradicts reports of other research on Phentermine where improvements in lipid profile were observed [13,25]. The findings of weight reduction without significant improvement in lipid profile for both anti-obesity medications may be a result of the short duration of the current study. Studies with longer duration would be beneficial in this regard. As for the lipid profile within the surgical group, both anti-obesity medications showed no significant change at all levels of TWL%. However, analyzing the subgroup of patients who achieved clinically significant TWL% (≥5%) showed that Lorcaserin resulted in greater reduction of TC and LDL compared to Phentermine (0.85 ± 0.51 vs. 0.02 ± 0.33 mmol/l, P = 0.003 and 0.57 ± 0.39 vs. 0.02 ± 0.25 mmol/l, P = 0.008, respectively). These findings support that modest weight loss [≥ 5% Total Weight (TWL%)] may improve obesity-related comorbidities [3,4].

As for glycemic control in the non-surgical group, at 3 months, both anti-obesity medications showed no significant differences in FBS and HbA1c levels. Such lack of glycemic improvement for Phentermine agrees with other studies where phentermine did not improve FBG at 3 months post treatment [13]. However, our observation of the lack of glycemic improvement for Lorcaserin is not consistent with other research probably due to their large sample of prediabetic (n = 6136) and diabetic (n = 351) patients [15,29]. A larger sample size may have allowed the current study to detect such findings. In terms of glycemic control in the surgical group, both Phentermine and Lorcaserin patients exhibited no significant improvements in FBS and HBA1C. This is supportive of a study where Phentermine resulted in no change in the number of diabetes medications suggesting no clinical improvement (albeit without measuring biochemical data e.g., HBA1c) [8]. To the best of our knowledge, no data exists on Lorcaserin's effect on cardiovascular risk factors among bariatric surgical patients with weight regain. Future research should address this gap as well as the underlying physiology.

In terms of side effects, a greater percentage of Phentermine patients reported side effects when compared to Lorcaserin patients (18 vs. 9.9%). Lorcaserin was generally well tolerated, with minimal side effects (most frequently headache 5.9%) in agreement with other research [15]. As for Phentermine, palpitations and insomnia were the most common, agreeing with other papers that reported insomnia as the most common side effect [13]. These findings are therefore consistent with this class of sympathomimetic drugs, where central excitation may manifest clinically as insomnia or increased heart rate [30]. This has contributed to the limited use of Phentermine among cardiovascular disease patients [10].

Cost data should be considered when selecting anti-obesity medications. In countries where anti-obesity medications are not covered by medical insurance, and drug cost is an expensive burden on patients, Phentermine may be a cheaper alternative to other expensive anti-obesity medications [28]. In countries with Universal health coverage, the high costs of some anti-obesity medications could burden the system. It is important to note that Phentermine is approved only for short-term use and probably not ideal for the management of a chronic condition such as obesity where the discontinuation of treatment might lead to weight regain. However, Phentermine intermittent and continuous therapies may result in the same weight loss accordingly, Phentermine intermittent regimens would be equally effective and would comply with the prescribing guidelines [21]. In contrast, Lorcaserin, although more expensive, is approved for long-term treatment of obesity.

This study has limitations. The relatively small sample size was because many patients were on combination pharmacotherapy (hence excluded) or due to unavailable data which is a limitation of retrospective studies. A longer follow-up would have enabled the current study to assess weight maintenance beyond 3 months. Moreover, reviewing blood pressure values as an indicator of cardiovascular risk would have been useful. The nutritional outcomes of anti-obesity medications (e.g. on hunger, food craving and satiety scores) were not assessed which are important factors in predicting weight gain. Likewise, assessment of the broader psych-behavioral, demographic, and environmental parameters could have shed more light on the characteristics associated with weight loss [29]. Future research would benefit from exploring these limitations. Nevertheless, the study has many strengths and has addressed identified gaps in the literature; we assessed the effectiveness and safety of Phentermine vs. Lorcaserin in real world practices, among obese non-surgical and surgical patients, using a wide range of anthropometric, lipid, glycemic, side effects and cost data. Such holistic approach has been less explored and studies pertaining to this variety of outcomes remain very limited.

## 5 Conclusion

At 3 months, among the non-surgical group, Phentermine patients had greater percentage of total weight loss (TWL%), and greater BMI reduction than Lorcaserin. Within the surgical group, Lorcaserin patients had significantly smaller TWL%, and BMI reduction than Phentermine. Among non-surgical and surgical patients, weight loss of ≥20% was only achieved with Phentermine. Lorcaserin exhibited significant TC and LDL improvements only among surgical patients with significant weight reduction (≥5% TW). Both medications were not associated with any glycemic (FBS, HbA1c) improvements among non-surgical and surgical patients. Using Phentermine instead of Lorcaserin when there is no contraindication could result in large savings, albeit with slightly more side effects. For non-surgical patients, using anti-obesity medications as part of a comprehensive multidisciplinary medical weight management program can result in weight loss. For surgical patients, anti-obesity medications can halt or reverse weight regain.

## Conflicts of interest

No conflicts of interest.

## Sources of funding

No sources of funding.

## Ethical approval

The medical research centre at hamad medical corporation (hmc) approved this retrospective study (irb, protocol #17193/17).

Ethical approval provided by The Medical Research Centre at Hamad Medical Corporation (HMC)(IRB, Protocol #17193/17).

## Author contribution

WE and WEA designed the study and interpreted the data and wrote the manuscript. Sr, me and im collected the data and contributed to the editing of the manuscript. All authors approved the final manuscript before submission.

## Research registration unique identifying number (uin)

Researchregistry4801.

## Guarantor

Walid El Ansari.

## Funding

This work was not supported by any funding, and no funding body was involved with the design of the study; collection, analysis, and interpretation of data; or with any writing for this manuscript.

## Provenance and peer review

Not commissioned, internally reviewed.
